# Which indicator should be used? A comparison between the incidence and intensity of catastrophic health expenditure: using difference-in-difference analysis

**DOI:** 10.1186/s13561-022-00403-w

**Published:** 2022-11-11

**Authors:** Jun Hyuk Koo, Hyun Woo Jung

**Affiliations:** 1 Yonsei University Wonju Industry-Academic Cooperation Foundation, Wonju, South Korea; 2 Department of Health Administration, Graduate School BK21 Graduate Program of Developing Glocal Experts in Health Policy and Management, Yonsei University, Wonju, South Korea

**Keywords:** Catastrophic health expenditure, Health policy, Out-of-pocket medical expenditures, Difference-in-difference analysis, Propensity score matching

## Abstract

**Background:**

Catastrophic health expenditure (CHE) represents out-of-pocket payment as a share of household income. Most previous studies have focused on incidence aspects when assessing health policy effects. However, because CHE incidence is a binary variable, the effect of the health policy could not accurately be evaluated. On the contrary, the intensity of CHE is a continuous variable that can yield completely different results from previous studies. This study reassesses the coverage expansion plan for four serious diseases using the intensity of CHE in Korea.

**Methods:**

We used the Korea Health Panel Study from 2013 to 2015 to conduct the analysis. The study population is households with chronic diseases patients. We divided the population into two groups: the policy beneficiary group, i.e., households with a patient of any of the four serious diseases, and the non-beneficiary group. A difference-in-difference model was employed to compare the variation in the intensity and incidence of CHE between the two groups. We defined the incidence of CHE as when the ratio of out-of-pocket medical expenses to household income is more than a threshold of 10%, and the intensity of CHE is the height of the ratio subtracting the threshold 10%.

**Results:**

The increased rate of CHE intensity in households with four serious diseases was lower than that in households with other chronic diseases. The interaction term, which represents the effect of the policy, has a significant impact on the intensity but not on the incidence of CHE.

**Conclusions:**

CHE indicators should be applied differently according to the purpose of policy evaluation. The incidence of CHE should be used as the final achievement indicator, and the intensity of CHE should be used as the process indicator. Furthermore, because CHE has an inherent characteristic that is measured by the ratio of household income to medical expenses, to lower this, a differential out-of-pocket maximum policy for each income class is more appropriate than a policy for strengthening the coverage for specific diseases.

**Supplementary Information:**

The online version contains supplementary material available at 10.1186/s13561-022-00403-w.

## Background

Catastrophic Health Expenditure (CHE) incidence is the proportion of households in which the ratio of out-of-pocket (OOP) medical expenses to their payment ability exceeds a certain threshold in the whole population [1–4]. Because the indicator has the advantage of collectively measuring the medical cost burden of households, it can be a useful tool for comparing healthcare coverage among countries. Thus, even the World Health Organization and the World Bank have adopted it as an indicator of financial risk protection [1]. Many researchers have used this indicator to evaluate the effect of health policy or systems that aim to lower the medical cost burden of patients [5–12].

However, recent studies have pointed out the significant inherent limitations of the indicator. Jung and Lee [13] conducted a systematic review of CHE, reviewing the time series trends of reported incidence and intensity of CHE in 34 countries. Intensity is a paired indicator with incidence [2], although it has not been used as much as incidence in previous studies. It measures the ratio of OOP medical expenses to the ability to pay (more accurately, it subtracts a certain threshold from the ratio). Jung and Lee [13] confirmed that CHE incidence in most countries does not significantly fluctuate in the time series, whereas the intensity is flexible. Furthermore, they reviewed four studies that employed difference-in-difference (DID) analysis to evaluate the healthcare system’s newly launched coverage expansion plan in different countries and confirmed that only one study found a significant decrease in CHE. In fact, many studies in Korea that assessed the effect of the benefit expansion policy of National Health Insurance (NHI) using the DID test concluded that there was a statistically significant effect on medical expenditure but no effect on CHE incidence [14–18]. Consequently, Jung and Lee [13] argued that incidence has an inherent limitation—it does not change and cannot sensitively reflect the policy effect. Additionally, they proposed intensity as an alternative indicator.

However, because Jung and Lee [13] conducted a systematic review, they could not demonstrate whether the reason why most DID analysis results did not reveal statistically significant policy effects was due to the intrinsic characteristics of CHE incidence. Moreover, they could not confirm whether the intensity of CHE can lead to opposite results when CHE incidence is used. Therefore, this study theoretically explains why CHE incidence inherently does not vary in the time series but intensity does.

**Fig. 1:**
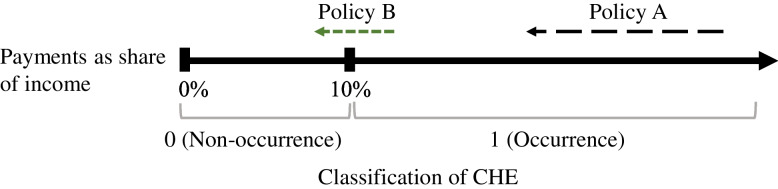
Limitation of using the incidence of CHE to analyze the policy effect

CHE incidence is a dichotomous variable. Therefore, even if the policy reduces the OOP medical expenses, households could still incur CHE when the proportion of OOP expenditure in household income exceeds the threshold. The reduced medical spending could not be reflected in the result, except for households that are close to the threshold. Figure 1 depicts that if policy effectiveness is determined only based on CHE incidence, the results of the analysis could be completely reversed, although Policy A is more effective than Policy B. However, because the intensity of CHE is a continuous variable, it is free from this problem.

This study contributes to the literature by demonstrating the arguments suggested by Jung and Lee [13] that results can vary depending on whether incidence or intensity is used when analyzing policy effect by conducting the DID test.

Many studies conducted propensity score matching (PSM) and DID tests to analyze the benefit expansion plan of NHI in Korea. They all reported no policy effect based on the incidence of CHE [16–20]. Since 2005, the Korean government has tried to strengthen the coverage of NHI for cancer and gradually extended the coverage by including other diseases (cardiovascular, cerebrovascular, and rare/intractable diseases). Coverage strengthening started by lowering the statutory copayment rate for patients with cancer from 20 to 10% in September 2005 and further reducing it to 5% in 2009 [21]. Additionally, the NHI reduced the copayment rate to 5–10% for rare and intractable, heart, and cerebrovascular diseases [21].

The problem was that the services not covered by the NHI were not captured in the statutory copayment rate. These services, such as magnetic resonance imaging, computerized tomography scans, chiropractic, and genetic testing for cancer treatment, are out of the NHI’s scope [22]. Such services are not subject to the fee regulation of the NHI but are set by medical providers, so they are likely to become expensive.

Therefore, the Korean government announced a comprehensive benefit expansion policy, “the benefit enhancement plan for four major diseases,” in June 2013 and implemented it in October [22, 23]. The policy allowed the NHI to cover the previously non-covered 25 items, including ultrasonic diagnostic tests and magnetic resonance imaging for heart diseases, in 2013, 100 in 2014, and 258 in 2015 [22].

This study evaluates the NHI’s benefit expansion policy for four serious diseases in Korea by using the incidence and intensity of the CHE indices and demonstrates that the results can be different between the two indicators. If the policy is found to be effective when the intensity of the CHE index is used, we can argue that the consistent negative results of the existing studies that analyzed the policy effect are due to the insufficiency of measurement methods.

## Methods

### Data sources

This study uses the Korea Health Panel Study (KHPS) version 1.5 database, which is jointly conducted by the Korea Institute for Health and Social Affairs and the National Health Insurance Service. Since 2008, the study has been conducted annually to analyze medical use behavior and expenditure in Korea. The KHPS is an official statistical database with a nationally representative sample extracted through a two-stage stratified cluster sampling process with probability proportionality based on the Korea Population and Housing Census. The KHPS includes healthcare utilization and expenditure based on the receipts of healthcare expenses and housekeeping books. Each healthcare expenditure is divided into three parts: copayment, insurance benefit for insured services, and uncovered services. In 2008, the KHPS started with 7866 households (24,616 individuals), and in 2012, the retention rate of the original sample was 70.6%. In 2013, 2222 households (6454 individuals) were added to the original sample, and 6983 households (18,834 individuals) were surveyed in 2015.

### Study population

This study analyzes the policy effect of the benefit expansion for four serious diseases in 2014. Some studies in Korea consider 2013 as the year when the policy was introduced and compare the period before 2012 and after 2014 [16–21]; however, the policy was implemented in October 2013, after the third quarter. Moreover, only 25 items were newly covered. Therefore, we believe it is insufficient to analyze the policy effect with 2013 as the base year. In fact, studies that used 2013 as the base year had inconsistent results [24]. Conversely, studies that used 2014 as the base year reported significant policy effects [25].

**Fig. 2:**
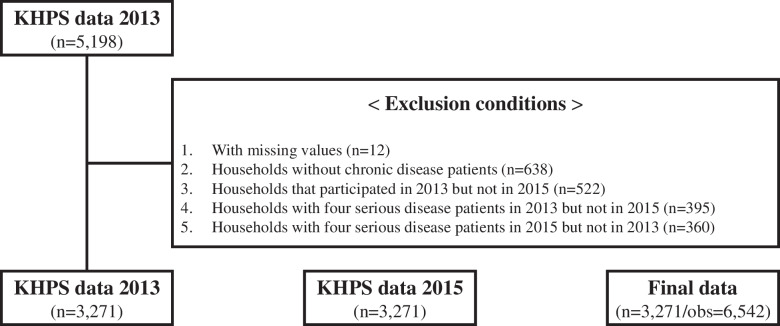
Sampling process

Therefore, we selected households with patients of chronic diseases in 2013 and 2015 as the study population. The policy beneficiary group (treatment group) is defined as a household with a member who has used medical care (emergency room, outpatient, or inpatient) for any of the four serious diseases at least once a year in 2013 and 2015. The four types of diseases were announced by the Ministry of Health and Welfare in Korea. We converted them into the international classification of diseases and presented the results as supplementary material. Figure 2 depicts the specific sampling process.

### Independent variables

The independent variables are based on Andersen’s health care utilization model [26], and we separate householder and household levels. The KHPS defines the head of a household as a householder and otherwise as a nonhouseholder. The householders’ characteristics include gender, elderly, education level, spouse, and whether they are economically active. The households’ characteristics include living alone, equivalized income, private health insurance, NHI, presence of disabled persons, and presence of any of the four serious diseases. The independent variables are shown in Table 1.

**Table 1:** Variable lists

**Independent variables**		**Coding**
**Householders’** **characteristics**	Gender	0: Male; 1: Female
	Elderly	0: No; 1: Yes (65+)
	Education level	0: Middle school or less; 1: High school or more
	Presence of a spouse	0: No; 1: Yes
	Economically active	0: No; 1: Yes
**Households’** **characteristics**	Living alone	0: No; 1: Yes
	Equivalized income	1: 1st quintile (the poorest); 2: 2nd quintile; 3: 3rd quintile; 4: 4th quintile; 5: 5th quintile (the richest)
	Private health insurance	0: No, 1: Yes
	National health insurance	0: No (Medical aid beneficiaries); 1: Yes
	Presence of disabled persons	0: No; 1: Yes
	Presence of persons with any of four serious diseases (= treatment)	0: No (Control group); 1: Yes (Treatment group)
**Dependent variables**		**Coding**
Indicator types of CHE	Incidence	*if OOP*/*HI* > 0.1, 1; otherwise, 0
	Intensity	*OOP*/*HI* − 0.1

*CHE* is catastrophic health expenditure; *OOP* is out-of-pocket payment; *HI* is household’s income

### Dependent variable

The dependent variables are the intensity and incidence of CHE, which is incurred when the proportion of OOP medical expenses in household income exceeds a certain threshold. In this study, we use a household’s total income to measure their ability to pay. The definition of OOP medical expenses varies depending on the characteristics of the data sources. In the KHPS, it is common to include only direct medical expenses, which are the minimum expenses incurred based on doctors’ opinions [27]. OOP medical expenses include medical and drug costs incurred on utilizing healthcare in the emergency room as well as outpatient and inpatient services. We set the CHE threshold level at 10%, which is the lowest value suggested by Wagstaff and van Doorslaer [2]. They suggested threshold values ranging from 10 to 40% when defining the ability to pay as total household income. Nevertheless, we chose 10% because many recent empirical and review studies suggested it as a representative threshold [28]. Moreover, if the threshold is set too high, the height of intensity and number of households with CHE will be too small, thereby making it insufficient for analysis. we calculated the incidence and intensity of CHE based on the above definitions of OOP medical expenses, the household’s ability to pay, and threshold level of CHE. The calculation is presented in the Table 1.

### Statistical analysis

We used the paired t-test and DID analysis to identify the effectiveness of the policy. Particularly, we performed the DID test to confirm the health policy effects by estimating two models. One model used CHE incidence as the dependent variable, and the other used CHE intensity. The two models were set to compare the characteristics of the two indicators. Both models include the time before and after the policy was implemented and whether the group is a policy beneficiary as follows:

$${Y}_i={\beta}_0+{\beta}_1T+{\beta}_2{P}_i+{\beta}_3T{P}_i+{\beta}_4{X}_i+{\varepsilon}_i$$

*Y*_*i*_ could be the incidence of CHE or refer to the intensity of CHE; *T*, the time variable comprises 0 and 1, representing 2013 and 2015, respectively. *P*_*i*_, the policy variable also comprises 0 and 1, indicating whether a group is a policy beneficiary (households with patients of four serious diseases) or not, respectively. *X*_*i*_ is the covariate variable. Before testing the policy effectiveness, we conducted a PSM and balancing test of the covariates to determine the similarity between the treatment and control groups. It uses the conditional probability of participating in the program under certain observable variables [29]. For its beneficial practical implications, PSM has gained popularity in evaluating healthcare programs [30, 31], health finance and insurance schemes [32], and many other areas of evaluation research. Rosenbaum and Rubin [29] suggested the covariate balance test because matching is based on propensity scores but not on covariates themselves. All analyses were performed using the Stata/SE version 16.0 software (Stata Corp., Texas, USA).

## Results

### General characteristics

Table 2 shows the general characteristics of the treatment and control groups in 2013. We found significant relationships between treatment and all variables, except living alone, through the bivariate analyses.

**Table 2:** General characteristics of households before treatment

Variables		Treatment (***N*** = 1145)	Control (***N*** = 2126)	Χ^**2**^ / t
Gender	Male	937 (81.83)	1649 (77.56)	8.20^**^
	Female	208 (18.17)	477 (22.44)	
Elderly	No	458 (40.00)	1461 (68.72)	253.15^***^
	Yes	687 (60.00)	665 (31.28)	
Education level	Middle school or less	591 (51.62)	809 (38.05)	55.92^***^
	High school or more	554 (48.38)	1317 (61.95)	
Presence of a spouse	No	264 (23.06)	558 (26.25)	4.02^*^
	Yes	881 (76.94)	1568 (73.75)	
Economically active	No	539 (47.07)	498 (23.42)	192.14^***^
	Yes	606 (52.93)	1628 (76.58)	
Living alone	No	956 (83.49)	1763 (82.93)	0.17
	Yes	189 (16.51)	363 (17.07)	
Equivalent income	1st quintile	323 (28.21)	394 (18.53)	80.40^***^
	2nd quintile	297 (25.94)	457 (21.50)	
	3rd quintile	229 (20.00)	428 (20.13)	
	4th quintile	152 (13.28)	458 (21.54)	
	5th quintile	144 (12.58)	389 (18.30)	
Private health insurance	No	390 (34.06)	415 (19.52)	84.81^***^
	Yes	755 (65.94)	1711 (80.48)	
National health insurance	No	1039 (90.74)	2034 (95.67)	31.81^***^
	Yes	106 (9.26)	92 (4.33)	
Presence of disabled persons	No	929 (81.14)	1965 (92.43)	93.05^***^
	Yes	216 (18.86)	161 (7.57)	
Incidence of CHE	No	735 (64.19)	1884 (88.62)	278.19^***^
	Yes	410 (35.81)	242 (11.38)	
Intensity of CHE		0.122 ± 0.005	0.049 ± 0.002	−13.08^***^

Values are presented as frequency (%) or mean ± standard deviation

### PSM and covariate balance test

We conducted PSM with replacement and checked the balance of covariates. We used the Stata command “teffects psmatch” and option “nn(2).” A balance between the treatment and control groups for each variable was tested using absolute standardized differences. Although there is no officially agreed-upon threshold level, an absolute standardized difference of < 0.1 has been considered a good balance of covariates [33]. The result of the balance test reveals that all covariates’ absolute standardized differences are less than 0.1 (Table 3).

**Table 3:** Covariate balance test after propensity score matching

	Standardized differences		Variance ratio	
	Raw	Matched	Raw	Matched
Gender (ref = Male)				
Female	−0.1059	−0.0389	0.8552	0.9437
Elderly (ref = No)				
Yes	0.6012	−0.0068	1.1173	0.9976
Education level (ref = Middle school or less)				
High school or more	−0.2761	0.0378	1.0598	0.9888
Presence of a spouse (ref = No)				
Yes	0.0736	0.0476	0.9174	0.9434
Economically active (ref = No)				
Yes	−0.5098	0.0203	1.3894	0.9834
Living alone (ref = No)				
Yes	−0.0148	0.0061	0.9744	1.0110
Equivalized income (ref = 1st quintile)				
2nd quintile	0.1051	−0.0302	1.1395	0.9630
3rd quintile	−0.0029	0.0121	0.9962	1.0183
4th quintile	−0.2189	0.0295	0.6819	1.0491
5th quintile	−0.1585	−0.0187	0.7363	0.9662
Private health insurance (ref = No)				
Yes	−0.3315	−0.0078	1.4291	1.0090
National health insurance (ref = No)				
Yes	0.1971	−0.0056	2.0315	0.9789
Presence of disabled persons (ref = No)				
Yes	0.3362	0.0091	2.1813	1.0230

### Paired t-test

Table 4 presents the results of the paired t-test of OOP medical expenses and household income after PSM. The analysis results reveal that there was an increase in OOP medical expenses in the non-beneficiary group from 2013 to 2015.

**Table 4:** Paired t-test

	2013	2015	Mean diff	t
OOP				
Total	201.55 ± 242.82	206.12 ± 266.52	4.57	0.55
Treatment	234.09 ± 259.05	232.84 ± 280.25	−1.25	− 0.12
Control	100.04 ± 141.47	122.74 ± 196.36	22.70	2.03^*^
Household income				
Total	3071.16 ± 3142.24	3052.22 ± 2864.47	− 18.94	− 0.38
Treatment	3136.96 ± 2789.89	3185.58 ± 3028.96	48.62	0.83
Control	2865.88 ± 4047.00	2636.15 ± 2228.93	− 229.73	−1.29

Values are presented as mean ± standard deviation, and the unit is 10,000 Won (Korean currency)

^*^ *p* < .05 ^**^ *p* < .01 ^***^ *p* < .001

### Analyzing policy effects on CHE using DID

As we mentioned above, there are two dependent variables in this study (intensity of CHE and incidence of CHE). Table 5 presents the results of the DID analyses for each dependent variable. After analyzing the effectiveness of the policy intervention while controlling the characteristics of households and householders, we found that the results of each group are completely different. The interaction term, which reveals the pure effect of the policy, has a significant impact on the intensity of CHE but not on its incidence.

**Table 5:** Difference-in-difference analyses

	Intensity of CHE Coef. (95% C.I.)		Incidence of CHE O.R. (95% C.I.)	
	Crude	Full	Crude	Full
Year (ref = 2013)				
2015	0.034^*^ (0.007, 0.062)	0.025 (−0.001, 0.051)	1.342 (0.909, 1.982)	1.193 (0.784, 1.814)
Treatment (ref = Control group)				
Treatment group	0.069^***^ (0.046, 0.091)	0.064^***^ (0.043, 0.086)	3.233^***^ (2.364, 4.422)	3.699^***^ (2.629, 5.205)
Year * Treatment	−0.034^*^ (− 0.065, − 0.002)	− 0.033^*^ (− 0.062, − 0.003)	0.742 (0.485, 1.136)	0.722 (0.456, 1.145)
Gender (ref = Male)				
Female		−0.004 (− 0.028, 0.019)		0.898 (0.641, 1.258)
Elderly (ref = No)				
Yes		0.016 (−0.000, 0.032)		1.466^**^ (1.169, 1.838)
Education level (ref = Middle school or less)				
High school or more		−0.003 (− 0.018, 0.011)		0.873 (0.716, 1.065)
Presence of a spouse (ref = No)				
Yes		0.022 (−0.003, 0.046)		1.669^**^ (1.161, 2.399)
Economically active (ref = No)				
Yes		−0.022^**^ (− 0.037, − 0.007)		0.797 (0.653, 0.973)
Living alone (ref = No)				
Yes		0.019 (−0.005, 0.043)		1.585^*^(1.115, 2.252)
Equivalized income (ref = 1st quintile)				
2nd quintile		−0.091^***^ (− 0.109, − 0.072)		0.373^***^ (0.297, 0.468)
3rd quintile		−0.124^***^ (− 0.146, − 0.103)		0.214^***^ (0.161, 0.283)
4th quintile		−0.146^***^ (− 0.170, − 0.122)		0.092^***^ (0.063, 0.133)
5th quintile		−0.163^***^ (− 0.189, − 0.138)		0.037^***^ (0.022, 0.064)
Private health insurance (ref = No)				
Yes		0.005 (−0.010, 0.021)		1.115 (0.909, 1.367)
National health insurance (ref = No)				
Yes		−0.101^***^(− 0.125, − 0.076)		0.186^***^ (0.132, 0.262)
Presence of disabled persons (ref = No)				
Yes		0.012 (−0.005, 0.029)		1.147 (0.917, 1.436)

*Coef* represents the coefficient of each variable, and *O.R.* represents the odds ratio of each variable

^*^ *p* < .05 ^**^ *p* < .01 ^***^ *p* < .001

## Discussion

This study aims to demonstrate that the policy effect result of the DID test can differ between the incidence and intensity indicators. For empirical evidence, we analyzed the case of the Korean policy—the 2014 benefit enhancement plan for four serious diseases.

Table 5 indicates that the absolute value of CHE intensity in the policy beneficiary group is higher than in the non-beneficiary group; however, after the policy, the gap in the intensity of CHE between the two groups has decreased, thereby implying that policy effects exist. This result differs from that of previous studies, which used the incidence of CHE as a dependent variable [16–18, 21, 25]. Furthermore, CHE incidence reveals no policy effect as expected. This finding supports Jung and Lee [13] that CHE incidence does not change in nature and cannot sensitively reflect the policy effect, and intensity can be an alternative indicator.

We can infer that the intensity of CHE is less affected by predisposition factors than the incidence of CHE (Table 5). These results reveal that intensity is a more appropriate indicator than incidence when estimating the level of financial burden of households. Because the unit of incidence is the ratio of the number of households to the whole population, it varies when more people are sick, even without any change in medical prices. Conversely, intensity is the ratio of OOP medical expenses to payment ability among households that incurred CHE incidence. Therefore, choosing intensity when conducting a microscopic study that analyzes the effect of a specific policy and/or considering the time series variation is appropriate.

Notably, assessing the effectiveness of the policy using only the intensity of CHE is insufficient because it is a sensitive indicator that can be interpreted as “effective” even with a slight reduction in OOP medical expenses. Because the intensity is a continuous variable, there is no standard for how much intensity should be reduced to be determined as having a policy effect. Conversely, because the incidence of CHE is measured as a dichotomous variable based on a specified threshold, we can determine whether the policy is effective using the threshold. Therefore, it is essential to distinguish between the variables when assessing the policy to expand health insurance coverage. When the NHI aims to eliminate households with CHE, the reduction in CHE incidence can be a final performance indicator, and the reduction in CHE intensity can be used as an intermediate indicator that determines proximity to the goal.

It is necessary to grasp the policy effect from various perspectives, apart from determining whether the policy intended to strengthen the coverage of some diseases is desirable. Thus, it is important to not only focus on CHE incidence but also analyze how much burden of medical expenses has been reduced even though CHE is already incurred. After considering the intensity of CHE, it is possible to interpret that the burden of medical costs is alleviated.

Furthermore, we discovered two unexpected essential results. First, focusing only on CHE would ignore the context of the policy. The benefit enhancement policy we analyzed aims to reduce the OOP burden of households with a patient of any of the four serious diseases in Korea. To confirm the policy effect in this context, the medical cost of the beneficiary group should be less than that of the non-beneficiary group. However, as Table 4 indicates, the OOP medical expenses in the treatment group did not decrease. Instead, it increased in the non-beneficiary group. Therefore, the results of the DID test in Table 5 can be due to the increase in the OOP medical expenses of the non-beneficiary group and not due to the decrease in the OOP medical expenses of the beneficiary group. It can be interpreted that the OOP medical expenses burden of households with a patient of any of the four serious diseases was transferred to households with other diseases. If the government tries to control the medical costs of some specific diseases, medical institutions can increase the cost of other diseases. Thus, the basic statistics for medical expenses are important when using CHE indicators.

Second, regardless of incidence or intensity, CHE is a combination of two variables—medical expenses and payment ability. Although there was no statistically significant variance in household income between 2013 and 2015, that of the control group decreased substantially (Table 4), which can affect the value of CHE. Jung and Lee [13] suggested that reducing CHE is only achieved by implementing policies that consider income levels, such as medical aid for the poor or a system that differentially charges medical expenses according to income levels.

The concept of CHE originated with the cost of serious diseases (catastrophic illness), which significantly interfere with life [34]. Havighurst, Blumstein, and Bovberjerg [35] argued that the policy should be altered to reduce the high medical costs that persist because economically burdensome diseases can be chronic diseases that require continuous treatment and rehabilitation. This argument has led to a trend that emphasizes the relative household income and medical expenses since the late 1970s [36, 37]. Feldstein [38] argued that the NHI should be designed to cover medical expenses of not more than 10% of annual household income, and the U.S. Congressional Budget Office proposed that this should be 15% [39]. Due to the inherent nature of CHE, which is measured by household income and medical expenses, a differential medical cost burden system based on the level of household income should be considered because it is difficult to reduce households with CHE under specific disease-related policies.

This study has a few limitations. First, we define the ability to pay as a household’s total income, which has the largest value among the options suggested by Wagstaff and van Doorslaer [2]. They defined the ability to pay as a household’s total income, household’s total income minus food expenses, total expenditure, or total expenditure minus food expenses. Therefore, the CHE might have been underestimated. Second, the PSM has limitations in that it splits the samples and reduces the size, thereby making it difficult to secure the sample size. Moreover, it makes room for sample bias. However, these limitations are manageable because the sample size after PSM is appropriate for this study and the sample bias was controlled again in the DID regression analysis. Third, the policy to strengthen the coverage of the four serious diseases began in 2005, but the KHPS data became available from 2008. Therefore, the period of the first stage of 2005–2008 was unavailable. Additionally, to analyze the exact policy effect using the DID method, the analysis period should be as short as possible so that the influence of other exogenous factors can be excluded to the maximum possible extent. Therefore, we set the analysis period as 2013, the last year of the second phase, and 2015, the year immediately following the third phase. Lastly, another limitation is that the four diseases were integrated and analyzed to divide the treatment and control groups.

## Conclusion

CHE has been widely used as a performance indicator to analyze the effectiveness of health policies in reducing the burden of medical expenses. However, incidence indicators are highly likely to be robust in policy assessment and are not sensitive to the effects of a reduction in OOP medical expense because they only detect changes around the threshold. However, previous studies usually relied on the incidence indicator. This study reevaluates the coverage expansion plan for four serious diseases in Korea using the intensity of CHE. Unlike the previous studies that used the incidence indicator, the results of this study confirm that there is a policy effect. This result is due to the difference in characteristics between the incidence and intensity of CHE. Therefore, policymakers should apply CHE indicators differently based on the purpose of policy evaluation. The incidence of CHE should be used as the final achievement indicator, and the intensity of CHE should be used as the process indicator. Furthermore, because CHE has an inherent characteristic, which is measured by the ratio of household income to medical expenses, to reduce it, a differential OOP maximum policy for each income class might be more appropriate than a policy to strengthen the coverage of specific diseases.

## Supplementary Information

**Additional file 1.**

## Data Availability

The datasets used during the current study are available from the corresponding author on reasonable request.

## Abbreviations

CHE: Catastrophic health expenditure
OOP: Out-of-pocket
KHPS: Korea Health Panel Study
DID: Difference-in-difference
NHI: National Health Insurance
PSM: Propensity score matching
