# Supplementary material for: Which indicator should be used? A comparison between the incidence and intensity of catastrophic health expenditure: using difference-in-difference analysis
Source: Health Econ Rev. 2022 Nov 11;12:58. doi: 10.1186/s13561-022-00403-w (PMC9650821; doi:10.1186/s13561-022-00403-w)
Supplement: Supplementary file 1 — Additional file 1. [file 13561_2022_403_MOESM1_ESM.docx]

Diseases list constituting the four diseases that targeted by the coverage expansion plan of Korean national health insurance.

1. **Cancer**

Table 1. List of cancer types covered by the coverage expansion policy

| C80 | Malignant neoplasm without specification of site |
| --- | --- |
| C00-C14 | Malignant neoplasms of lip, oral cavity and pharynx(C00-C14) |
| C15 | Malignant neoplasm of oesophagus |
| C16 | Malignant neoplasm of stomach |
| C18 | Malignant neoplasm of colon |
| C19 | Malignant neoplasm of rectosigmoid junction |
| C20 | Malignant neoplasm of rectum |
| C21 | Malignant neoplasm of anus and anal canal |
| C22 | Malignant neoplasm of liver and intrahepatic bile ducts |
| C23 | Malignant neoplasm of gallbladder |
| C24 | Malignant neoplasm of other and unspecified parts of biliary tract |
| C25 | Malignant neoplasm of pancreas |
| C30-C39 | Malignant neoplasms of respiratory and intrathoracic organs |
| C40 | Malignant neoplasm of bone and articular cartilage of limbs |
| C41 | Malignant neoplasm of bone and articular cartilage of other and unspecified sites |
| C43 | Malignant melanoma of skin of genital organs |
| C44 | Other malignant neoplasms of skin |
| C45-C49 | Malignant neoplasms of mesothelial and soft tissue |
| C50 | Malignant neoplasm of breast |
| C51-C58 | Malignant neoplasms of female genital organs |
| C61 | Malignant neoplasm of prostate |
| C62 | Malignant neoplasm of testis |
| C63 | Malignant neoplasm of other and unspecified male genital organs |
| C64 | Malignant neoplasm of kidney, except renal pelvis |
| C66 | Malignant neoplasm of ureteric orifice of bladder |
| C67 | Malignant neoplasm of bladder |
| C69 | Malignant neoplasm of eye and adnexa |
| C71 | Malignant neoplasm of brain |
| C72 | Malignant neoplasm of spinal cord, cranial nerves and other parts of central nervous system |
| C76-C80 | Malignant neoplasms of ill-defined, secondary and unspecified sites |
| C73 | Malignant neoplasm of thyroid gland |
| C81 | Hodgkin lymphoma |
| C85.9 | Non-Hodgkin lymphoma, unspecified |
| C95.9 | Leukaemia, unspecified |
| C96 | Other and unspecified malignant neoplasms of lymphoid, haematopoietic and related tissue |
| C81-C96 | Malignant neoplasms of lymphoid, haematopoietic and related tissue |
| C90 | Multiple myeloma and malignant plasma cell neoplasms |
| C91 | Lymphoid leukaemia |
| C92 | Myeloid leukaemia |
| C93 | Monocytic leukaemia |
| C94 | Other leukaemias of specified cell type |
| C95 | Leukaemia of unspecified cell type |

1. **Cardiovascular diseases**

Table 2. List of cardiovascular disease types covered by the coverage expansion policy

| I20-I25 | Ischaemic heart diseases |
| --- | --- |
| I30-I52 | Other forms of heart disease |

1. **Cerebrovascular diseases**

Table 3. List of Cerebrovascular disease types covered by the coverage expansion policy

| I64 | Stroke, not specified as haemorrhage or infarction |
| --- | --- |
| I61.9 | Intracerebral haemorrhage, unspecified |

1. **Rare/intractable disease**

Table 4. List of rare/intractable disease types covered by the coverage expansion policy

| A81.0 | Creutzfeldt-Jakob disease |
| --- | --- |
| D66 | Hereditary factor Ⅷ deficiency |
| D67 | Hereditary factor Ⅸ deficiency |
| D68.0 | Von Willebrand’s disease |
| D68.1 | Hereditary factor Ⅺ deficiency |
| D68.2 | Hereditary deficiency of other clotting factors |
| D55.0 | Anaemia due to glucose-6- phosphate dehydrogenase [G6PD] deficiency |
| D55.2 | Anaemia due to disorders of glycolytic enzymes |
| D56.0 | Alpha thalassaemia |
| D56.1 | Beta thalassaemia |
| D56.2 | Delta-beta thalassaemia |
| D56.3 | Thalassaemia trait |
| D56.4 | Hereditary persistence of fetal haemoglobin |
| D59.3 | Haemolytic-uraemic syndrome |
| D59.5 | Paroxysmal nocturnal haemoglobinuria |
| D60.0 | Chronic acquired pure red cell aplasia |
| D61.0 | Constitutional aplastic anaemia |
| D61.3 | Idiopathic aplastic anaemia |
| D61.9 | Aplastic anaemia, unspecified |
| D64.4 | Congenital dyserythropoietic anaemia |
| D68.6 | Other thrombophilia |
| D69.1 | Qualitative platelet defects |
| D69.30 | Evans’ syndrome |
| D70.8 | Other agranulocytosis |
| D71 | Functional disorders of polymorphonuclear neutrophils |
| D76.1 | Haemophagocytic lymphohistiocytosis |
| D76.3 | Other histiocytosis syndromes |
| D80.0 | Hereditary hypogammaglobulinaemia |
| D80.1 | Nonfamilial hypogammaglobulinaemia |
| D80.2 | Selective deficiency of immunoglobulin A[IgA] |
| D80.3 | Selective deficiency of immunoglobulin G [IgG] subclasses |
| D80.4 | Selective deficiency of immunoglobulin M[IgM] |
| D80.5 | Immunodeficiency with increased immunoglobulin M[IgM] |
| D80.6 | Antibody deficiency with near-normal immunoglobulins or with hyperimmunoglobulinaemia |
| D80.8 | Other immunodeficiencies with predominantly antibody defects |
| D81.0 | Severe combined immunodeficiency [SCID] with reticular dysgenesis |
| D81.1 | Severe combined immunodeficiency [SCID] with low T-and B-cell numbers |
| D81.2 | Severe combined immunodeficiency [SCID] with low or normal B-cell numbers |
| D81.3 | Adenosine deaminase[ADA] deficiency |
| D81.4 | Nezelof’s syndrome |
| D81.5 | Purine nucleoside phosphorylase[PNP] deficiency |
| D81.6 | Major histocompatibility complex class I deficiency |
| D81.7 | Major histocompatibility complex class Ⅱ deficiency |
| D81.8 | Other combined immunodeficiencies |
| D81.9 | Combined immunodeficiency, unspecified |
| D82.0 | Wiskott-Aldrich syndrome |
| D82.1 | Di George’s syndrome |
| D82.2 | Immunodeficiency with short-limbed stature |
| D82.3 | Immunodeficiency following hereditary defective response to Epstein-Barr virus |
| D82.4 | Hyperimmunoglobulin E [IgE] syndrome |
| D83.0 | Common variable immunodeficiency with predominant abnormalities of B-cell numbers and function |
| D83.1 | Common variable immunodeficiency with predominant immunoregulatory T-cell disorders |
| D83.2 | Common variable immunodeficiency with autoantibodies to B-or T-cells |
| D84.0 | Lymphocyte function antigen-1[LFA-1] defect |
| D84.1 | Defects in the complement system |
| D86.0 | Sarcoidosis of lung |
| D86.1 | Sarcoidosis of lymph nodes |
| D86.2 | Sarcoidosis of lung with sarcoidosis of lymph nodes |
| D86.3 | Sarcoidosis of skin |
| D86.8 | Sarcoidosis of other and combined sites |
| D89.1 | Cryoglobulinaemia |
| E22.0 | Acromegaly and pituitary gigantism |
| E23.0 | Hypopituitarism |
| E24.0 | Pituitary-dependent Cushing’s disease |
| E24.1 | Nelson’s syndrome |
| E24.3 | Ectopic ACTH syndrome |
| E25.0 | Congenital adrenogenital disorders associated with enzyme deficiency |
| E25.9 | Adrenogenital disorder, unspecified |
| E26.8 | Other hyperaldosteronism |
| E27.1 | Primary adrenocortical insufficiency |
| E27.2 | Addisonian crisis |
| E27.4 | Other and unspecified adrenocortical insufficiency |
| E34.8 | Other specified endocrine disorders |
| E55.0 | Rickets, active |
| E70.0 | Classical phenylketonuria |
| E70.1 | Other hyperphenylalaninaemias |
| E70.2 | Disorders of tyrosine metabolism |
| E70.3 | Albinism |
| E70.8 | Other disorders of aromatic amino-acid metabolism |
| E71.0 | Maple-syrup-urine disease |
| E71.1 | Other disorders of branched-chain amino-acid metabolism |
| E71.3 | Disorders of fatty-acid metabolism |
| E72.0 | Disorders of amino-acid transport |
| E72.1 | Disorders of sulfur-bearing amino-acid metabolism |
| E72.2 | Disorders of urea cycle metabolism |
| E72.3 | Disorders of lysine and hydroxylysine metabolism |
| E72.4 | Disorders of ornithine metabolism |
| E72.5 | Disorders of glycine metabolism |
| E72.8 | Other specified disorders of amino-acid metabolism |
| E73.0 | Congenital lactase deficiency |
| E74.0 | Glycogen storage disease |
| E74.2 | Disorders of galactose metabolism |
| E74.4 | Disorders of pyruvate metabolism and gluco- neogenesis |
| E74.8 | Other specified disorders of carbohydrate metabolism |
| E75.0 | GM₂-gangliosidosis |
| E75.1 | Other gangliosidosis |
| E75.2 | Other sphingolipidosis |
| E75.4 | Neuronal ceroid lipofuscinosis |
| E75.5 | Other lipid storage disorders |
| E76.0 | Mucopolysaccharidosis, type Ⅰ |
| E76.1 | Mucopolysaccharidosis, type Ⅱ |
| E76.2 | Other mucopolysaccharidoses |
| E77.0 | Defects in post-translational modification of lysosomal enzymes |
| E77.1 | Defects in glycoprotein degradation |
| E79.1 | Lesch-Nyhan syndrome |
| E80.2 | Other porphyria |
| E83.0 | Disorders of copper metabolism |
| E83.1 | Disorders of iron metabolism |
| E83.3 | Disorders of phosphorus metabolism and phosphatases |
| E84.0 | Cystic fibrosis with pulmonary manifestations |
| E84.1 | Cystic fibrosis with intestinal manifestations |
| E85.0 | Non-neuropathic heredofamilial amyloidosis |
| E85.1 | Neuropathic heredofamilial amyloidosis |
| E85.2 | Heredofamilial amyloidosis, unspecified |
| E85.4 | Organ-limited amyloidosis |
| F80.3 | Acquired aphasia with epilepsy [Landau-Kleffner] |
| F84.2 | Rett’s syndrome |
| G10 | Huntington’s disease |
| G11.0 | Congenital nonprogressive ataxia |
| G11.1 | Early-onset cerebellar ataxia with essential tremor |
| G11.2 | Late-onset cerebellar ataxia |
| G11.3 | Cerebellar ataxia with defective DNA repair |
| G11.4 | Hereditary spastic paraplegia |
| G11.8 | Other hereditary ataxias |
| G11.9 | Hereditary ataxia, unspecified |
| G12.0 | Infantile spinal muscular atrophy, type Ⅰ[Werdnig-Hoffman] |
| G12.1 | Other inherited spinal muscular atrophy |
| G12.2 | Motor neuron disease |
| G12.8 | Other spinal muscular atrophies and related syndromes |
| G12.9 | Spinal muscular atrophy, unspecified |
| G23.0 | Hallervorden-Spatz disease |
| G23.1 | Progressive supranuclear ophthalmoplegia [Steele-Richardson-Olszewski] |
| G31.81 | Leigh’s disease |
| G35 | Multiple sclerosis |
| G36.0 | Neuromyelitis optica[Devic] |
| G40.4 | Other generalized epilepsy and epileptic syndromes |
| G41.0 | Grand mal status epilepticus |
| G41.1 | Petit mal status epilepticus |
| G41.2 | Complex partial status epilepticus |
| G41.8 | Other status epilepticus |
| G41.9 | Status epilepticus, unspecified |
| G47.4 | Narcolepsy and cataplexy |
| G51.2 | Melkersson’s syndrome |
| G56.4 | Complex regional pain syndrome typeⅡ |
| G57.80 | Complex regional pain syndrome type II of lower limb |
| G60.0 | Hereditary motor and sensory neuropathy, types I-IV |
| G61.0 | Guillain-Barré syndrome |
| G61.8 | Other inflammatory polyneuropathies |
| G70.0 | Myasthenia gravis |
| G70.2 | Congenital and developmental myasthenia |
| G71.0 | Muscular dystrophy |
| G71.1 | Myotonic disorders |
| G71.2 | Congenital myopathies |
| G71.3 | Mitochondrial myopathy, NEC |
| G71.9 | Primary disorder of muscle, unspecified |
| G72.3 | Periodic paralysis |
| G73.1* | Lambert-Eaton syndrome(C00-D48†) |
| G95.0 | Syringomyelia and syringobulbia |
| H31.2 | Hereditary choroidal dystrophy |
| H35.01 | Exudative retinopathy |
| H35.51 | Pigmentary retinal dystrophy |
| H35.58 | Other dystrophies primarily involving the sensory retina |
| H35.59 | Unspecified hereditary retinal dystrophy |
| H49.8 | Other paralytic strabismus |
| I27.0 | Primary pulmonary hypertension |
| I27.8 | Other specified pulmonary heart diseases |
| I42.0 | Dilated cardiomyopathy |
| I42.1 | Obstructive hypertrophic cardiomyopathy |
| I42.2 | Other hypertrophic cardiomyopathy |
| I42.3 | Endomyocardial(eosinophilic) disease |
| I42.4 | Endocardial fibroelastosis |
| I49.8 | Other specified cardiac arrhythmias |
| I67.5 | Moyamoya disease |
| I73.1 | Thromboangiitis obliterans[Buerger] |
| I78.0 | Hereditary haemorrhagic telangiectasia |
| I82.0 | Budd-Chiari syndrome |
| J84.0 | Alveolar and parietoalveolar conditions) |
| J84.18 | Other interstitial pulmonary diseases with fibrosis |
| K50.0 | Crohn’s disease of small intestine |
| K50.1 | Crohn’s disease of large intestine |
| K50.8 | Other Crohn’s disease |
| K74.3 | Primary biliary cirrhosis |
| K75.4 | Autoimmune hepatitis |
| K83.0 | Cholangitis in choledocholithiasis(K80.3--K80.4-) |
| L10.0 | Pemphigus vulgaris |
| L10.2 | Pemphigus foliaceus |
| L12.0 | Bullous pemphigoid |
| L12.1 | Cicatricial pemphigoid |
| L12.3 | Acquired epidermolysis bullosa |
| M06.1 | Adult-onset Still’s disease |
| M08.0 | Juvenile rheumatoid arthritis with or without rheumatoid factor |
| M08.1 | Juvenile ankylosing spondylitis |
| M08.2 | Juvenile arthritis with systemic onset |
| M08.3 | Juvenile polyarthritis (seronegative) |
| M30.0 | Polyarteritis nodosa |
| M30.1 | Polyarteritis with lung involvement [Churg- Strauss] |
| M30.2 | Juvenile polyarteritis |
| M31.0 | Hypersensitivity angiitis |
| M31.1 | Thrombotic microangiopathy |
| M31.2 | Lethal midline granuloma |
| M31.3 | Wegener’s granulomatosis |
| M31.4 | Aortic arch syndrome [Takayasu] |
| M31.7 | Microscopic polyangiitis |
| M32.1 | Systemic lupus erythematosus with organ or system involvement |
| M33.0 | Juvenile dermatomyositis |
| M33.1 | Other dermatomyositis |
| M33.2 | Polymyositis |
| M34.0 | Progressive systemic sclerosis |
| M34.1 | CR(E)ST syndrome |
| M34.8 | Other forms of systemic sclerosis |
| M35.0 | Sicca syndrome[Sjögren] |
| M35.1 | Other overlap syndromes |
| M35.2 | Behçet’s disease |
| M35.3 | Polymyalgia rheumatica with giant cell arteritis(M31.5) |
| M35.4 | Diffuse(eosinophilic) fasciitis |
| M35.5 | Multifocal fibrosclerosis |
| M35.6 | Relapsing panniculitis[Weber-Christian] |
| M61.1 | Myositis ossificans progressiva |
| M88.0 | Paget’s disease of skull |
| M88.8 | Paget’s disease of other bones |
| M88.9 | Paget’s disease of bone, unspecified |
| M89.0 | Complex regional pain syndrome type I |
| M92.2 | Juvenile osteochondrosis of hand |
| M93.1 | Kienböck’s disease of adults |
| M94.1 | Relapsing polychondritis |
| N04.0 | Nephrotic syndrome with minor glomerular abnormality |
| N04.1 | Nephrotic syndrome with focal and segmental glomerular lesions |
| N04.2 | Nephrotic syndrome with diffuse membranous glomerulonephritis |
| N04.3 | Nephrotic syndrome with diffuse mesangial proliferative glomerulonephritis |
| N04.4 | Nephrotic syndrome with diffuse endocapillary proliferative glomerulonephritis |
| N04.5 | Nephrotic syndrome with diffuse mesangiocapillary glomerulonephritis |
| N04.6 | Nephrotic syndrome with dense deposit disease |
| N04.7 | Nephrotic syndrome with diffuse crescentic glomerulonephritis |
| N25.1 | Nephrogenic diabetes insipidus |
| Q03.1 | Atresia of foramina of Magendie and Luschka |
| Q04.3 | Other reduction deformities of brain |
| Q04.6 | Congenital cerebral cysts |
| Q05.0 | Cervical spina bifida with hydrocephalus |
| Q05.1 | Thoracic spina bifida with hydrocephalus |
| Q05.2 | Lumbar spina bifida with hydrocephalus |
| Q05.3 | Sacral spina bifida with hydrocephalus |
| Q05.4 | Unspecified spina bifida with hydrocephalus |
| Q05.5 | Cervical spina bifida without hydrocephalus |
| Q05.6 | Thoracic spina bifida without hydrocephalus |
| Q05.7 | Lumbar spina bifida without hydrocephalus |
| Q05.8 | Sacral spina bifida without hydrocephalus |
| Q05.9 | Spina bifida, unspecified |
| Q06.2 | Diastematomyelia |
| Q07.0 | Arnold-Chiari syndrome |
| Q17.2 | Microtia |
| Q20.0 | Common arterial trunk |
| Q20.1 | Double outlet right ventricle |
| Q20.2 | Double outlet left ventricle |
| Q20.3 | Discordant ventriculoarterial connection |
| Q20.4 | Double inlet ventricle |
| Q20.5 | Discordant atrioventricular connection |
| Q21.2 | Atrioventricular septal defect |
| Q21.3 | Tetralogy of Fallot |
| Q21.4 | Aortopulmonary septal defect |
| Q21.8 | Other congenital malformations of cardiac septa |
| Q22.0 | Pulmonary valve atresia |
| Q22.4 | Congenital tricuspid stenosis |
| Q22.5 | Ebstein’s anomaly |
| Q22.6 | Hypoplastic right heart syndrome |
| Q23.0 | Congenital stenosis of aortic valve |
| Q23.1 | Congenital insufficiency of aortic valve |
| Q23.2 | Congenital mitral stenosis |
| Q23.3 | Congenital mitral insufficiency |
| Q23.4 | Hypoplastic left heart syndrome |
| Q23.8 | Other congenital malformations of aortic and mitral valves |
| Q23.9 | Congenital malformation of aortic and mitral valves, unspecified |
| Q24.4 | Congenital subaortic stenosis |
| Q24.5 | Malformation of coronary vessels |
| Q24.6 | Congenital heart block |
| Q25.1 | Coarctation of aorta |
| Q25.2 | Atresia of aorta |
| Q25.3 | Stenosis of aorta |
| Q25.5 | Atresia of pulmonary artery |
| Q26.0 | Congenital stenosis of vena cava (inferior)(superior) |
| Q26.1 | Persistent left superior vena cava |
| Q26.2 | Total anomalous pulmonary venous connection |
| Q26.3 | Partial anomalous pulmonary venous connection |
| Q26.4 | Anomalous pulmonary venous connection, unspecified |
| Q26.5 | Anomalous portal venous connection |
| Q26.6 | Portal vein-hepatic artery fistula |
| Q38.3 | Other congenital malformations of tongue |
| Q44.2 | Atresia of bile ducts |
| Q61.1 | Polycystic kidney, autosomal recessive |
| Q64.1 | Exstrophy of urinary bladder |
| Q74.3 | Arthrogryposis multiplex congenita |
| Q75.0 | Craniosynostosis |
| Q75.1 | Craniofacial dysostosis |
| Q75.4 | Mandibulofacial dysostosis |
| Q77.0 | Achondrogenesis |
| Q77.1 | Thanatophoric short stature |
| Q77.2 | Short rib syndrome |
| Q77.3 | Chondrodysplasia punctata |
| Q77.4 | Achondroplasia |
| Q77.5 | Dystrophic dysplasia |
| Q77.6 | Chondroectodermal dyplasia |
| Q77.7 | Spondyloepiphyseal dysplasia |
| Q77.8 | Other osteochondrodysplasia with defects of growth of tubular bones and spine |
| Q77.9 | Osteochondrodysplasia with defects of growth of tubular bones and spine, unspecified |
| Q78.0 | Osteogenesis imperfecta |
| Q78.1 | Polyostotic fibrous dysplasia |
| Q78.2 | Osteopetrosis |
| Q78.3 | Progressive diaphyseal dysplasia |
| Q78.4 | Enchondromatosis |
| Q78.5 | Metaphyseal dysplasia |
| Q78.6 | Multiple congenital exostoses |
| Q79.0 | Congenital diaphragmatic hernia |
| Q79.1 | Other congenital malformations of diaphragm |
| Q79.2 | Exomphalos |
| Q79.3 | Gastroschisis |
| Q79.4 | Prune belly syndrome |
| Q79.5 | Other congenital malformations of abdominal wall |
| Q79.6 | Ehlers-Danlos syndrome |
| Q79.8 | Other congenital malformations of musculoskeletal system |
| Q79.9 | Congenital malformation of musculoskeletal system, unspecified |
| Q80.1 | X-linked ichthyosis; steroid sulfatase deficiency |
| Q80.4 | Harlequin fetus |
| Q81.1 | Epidermolysis bullosa letalis |
| Q81.2 | Epidermolysis bullosa dystrophica |
| Q85.0 | Neurofibromatosis (nonmalignant) |
| Q85.1 | Tuberous sclerosis |
| Q85.8 | Other phakomatoses, NEC |
| Q86.0 | Fetal alcohol syndrome (dysmorphic) |
| Q87.0 | Congenital malformation syndromes predominantly affecting facial appearance |
| Q87.1 | Congenital malformation syndromes predominantly associated with short stature |
| Q87.2 | Congenital malformation syndromes predominantly involving limbs |
| Q87.3 | Congenital malformation syndromes involving early overgrowth |
| Q87.4 | Marfan’s syndrome |
| Q87.8 | Other specified congenital malformation syndromes, NEC |
| Q90.0 | Trisomy 21, meiotic nondisjunction |
| Q90.1 | Trisomy 21, mosaicism (mitotic nondisjunction) |
| Q90.2 | Trisomy 21, translocation |
| Q90.9 | Down’s syndrome, unspecified |
| Q91.0 | Trisomy 18, meiotic nondisjunction |
| Q91.1 | Trisomy 18, mosaicism (mitotic nondisjunction) |
| Q91.2 | Trisomy 18, translocation |
| Q91.4 | Trisomy 13, meiotic nondisjunction |
| Q91.5 | Trisomy 13, mosaicism (mitotic nondisjunction) |
| Q91.6 | Trisomy 13, translocation |
| Q91.7 | Patau’s syndrome, unspecified |
| Q93.4 | Deletion of short arm of chromosome 5 |
| Q93.5 | Other deletions of part of a chromosome |
| Q96.0 | Karyotype 45, X |
| Q96.1 | Karyotype 46, X iso(Xq) |
| Q96.2 | Karyotype 46, X with abnormal sex chromosome, except iso(Xq) |
| Q96.3 | Mosaicism, 45, X/46, XX or XY |
| Q96.4 | Mosaicism, 45, X/other cell line(s) with abnormal sex chromosome |
| Q98.0 | Klinefelter’s syndrome karyotype 47, XXY |
| Q98.1 | Klinefelter’s syndrome, male with more than two X chromosomes |
| Q98.2 | Klinefelter’s syndrome, male with 46,XX karyotype |
| Q99.2 | Fragile X chromosome |
